# Learning Curves in Pediatric Robot-Assisted Pyeloplasty: A Systematic Review

**DOI:** 10.3390/jcm11236935

**Published:** 2022-11-24

**Authors:** Niklas Pakkasjärvi, Nellai Krishnan, Liisi Ripatti, Sachit Anand

**Affiliations:** 1 Department of Pediatric Surgery, Turku University Hospital, 20521 Turku, Finland; 2 Department of Pediatric Surgery, All India Institute of Medical Sciences, New Delhi 110029, India

**Keywords:** robot-assisted surgery, surgical learning, learning curve, pediatric urology, pyeloplasty

## Abstract

**Background**: Robot-assisted surgery demands a specific skillset of surgical knowledge, skills, and attitudes from the robotic surgeon to function as part of the robotic team and for maximal utility of the assistive surgical robot. Subsequently, the learning process of robot-assisted surgery entails new modes of learning. We sought to systematically summarize the published data on pediatric robot-assisted pyeloplasty (pRALP) to decipher the learning process by analyzing learning curves. **Methods**: This review followed the PRISMA guidelines. PubMed, EMBASE, Web of Science, and Scopus databases were systematically searched for ‘learning curve’ AND ‘pediatric pyeloplasty’. All studies presenting outcomes of learning curves (LC) in the context of pRALP in patients < 18 years of age were included. Studies comparing LC in pRALP versus open and/or laparoscopic pyeloplasty were also included; however, those solely focusing on LC in non-robotic approaches were excluded. The methodological quality was assessed using the Newcastle and Ottawa scale. **Results**: Competency was non-uniformly defined in all fifteen studies addressing learning curves in pRALP. pRALP was considered safe at all stages. Proficiency in pRALP was reached after 18 cases, while competency was estimated to demand 31 operated cases with operative duration as outcome variable. **Conclusions**: Pediatric RALP is safe during the learning process and ‘learning by doing’ improves efficiency. Competencies with broader implications than time must be defined for future studies.

## 1. Introduction

Robot-assisted surgery demands a specific skillset of surgical knowledge, skills, and attitudes from the robotic surgeon to function as part of the robotic team and for maximal utility of the assistive surgical robot. Subsequently, the learning process of robot-assisted surgery entails new modes of learning from the multi-professional team and the robotic surgeon.

Robot-assisted laparoscopic pyeloplasty (RALP) has become the standard operative intervention for hydronephrosis in children and adults [1]. While robot-assisted surgery presents many advantages over traditional laparoscopy through increased dexterity and improved and magnified optics, it has been criticized, especially from economic considerations [2]. Looking at hospital costs, irrespective of robotic acquisition costs, most aspects considered, robot-assisted pyeloplasty is feasible [3]. Pediatric RALP has also proven safe during the learning process [4], but the length of the learning process is open for debate with a range of numbers required for proficiency [5]. Laparoscopic pyeloplasty has all the benefits of minimally invasive surgery with similar costs to open surgery but is hampered by longer learning curves (LC) [6].

Technological progress is fast and will develop surgical robots and respective instruments in ways currently unimaginable. Still, the utilization of robot-assisted surgery in pediatric surgery fellowships has been slow [7]. Reasons for the slow incorporation have been attributed to factors of incomplete supportive evidence and increased operative times. Only 18% of program directors in the US believed that robotic training should be part of pediatric surgical fellowships previously. Perceptions of robot-assisted surgery are variable, with much of the hesitancy being dependent on misconception [8]. The tenets of robotic surgery are stronger within pediatric urology and the robotic platform offers a verified alternative operative platform for pediatric patients with positive future perspectives [9,10,11].

Training for robotic surgery must be methodological and efficient for continued progress. The process of surgical learning is often analyzed with LC [12]. Most commonly, the variable measured is time, and operative times are analyzed as a function of incremental operative experience. Many factors influence operative times and describing the progress of team-dependent operations with LC induces several possible confounding factors. We sought to systematically summarize the published data on pediatric RALP to decipher the learning process. Primary outcomes regarding LC were (i) how is competence assessed currently in pediatric RALP, and (ii) what is the timeline for learning, maintenance, and decline. Secondary outcomes assessed were the clarification of confounding factors of LC associated with pediatric RALP and outcomes in relation to LC.

## 2. Materials and Methods

### 2.1. Search Strategy

The literature search was conducted as per the Preferred Reporting Items for Systematic Reviews and Meta-Analyses (PRISMA) guidelines [13]. Two investigators (NK and SA) independently conducted searches on PubMed, EMBASE, Web of Science, and Scopus databases on the 5 September 2022. The search keywords used were (learning curve) AND (pediatric pyeloplasty) (“learning curve”[MeSH Terms]) OR (“learning”[All Fields] AND “curve”[All Fields]) OR “learning curve”[All Fields]) AND ((“paediatrics”[All Fields] OR “pediatrics”[MeSH Terms] OR “pediatrics”[All Fields] OR “paediatric”[All Fields] OR “pediatric”[All Fields]) AND (“pyeloplasties”[All Fields] OR “pyeloplasty”[All Fields])). The total search records were analyzed, and duplications were removed. Subsequently, the eligibility criteria were applied to screen the studies.

### 2.2. Eligibility Criteria

The inclusion criteria were all studies presenting outcomes of learning curves in the context of RALP procedure in patients aged less than 18 years. LC were to be formally presented in the eligible articles. Studies comparing LC in pRALP versus open and/or laparoscopic pyeloplasty were also included; however, those solely focusing on LC in non-robotic approaches were excluded. Case reports, literature reviews, commentaries, editorials, conference abstracts, and opinion articles were also excluded. Studies comparing LC of trainee surgeons with senior surgeons were excluded.

### 2.3. Data Extraction

Search results were obtained by two independent researchers (NK and SA). Extracted information included: first author’s name, publication year, article title, study period, study design, sample size, average age of the cohort, number of surgeons performing the procedures, and outcomes of the surgery. Any disagreements among the researchers were settled by consensus or discussion with the third author (NP). Data synthesis was independently performed by two investigators (NK and SA) using Microsoft Excel spreadsheets. The primary outcomes of this study were the method of assessment of LC, number of cases during the LC and complications during the LC. The secondary outcomes of the study were the role of confounding variables in LC, if any.

### 2.4. Quality Assessment

The Newcastle–Ottawa scale [14] was used for quality assessment of the included cohort studies. This validated scale assesses the methodological quality under three domains—selection, comparability, and outcome. A total of eight items are included in these domains. Two authors (SA and NK) independently assessed the methodological quality. Any dispute was resolved through consensus or by discussion with a third author (NP).

## 3. Results

**Figure 1:**
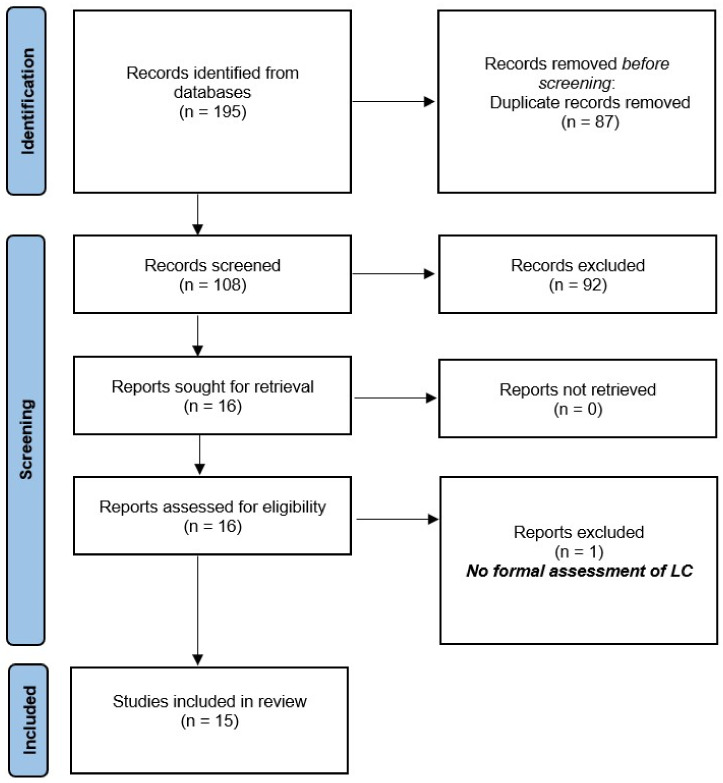
Selection of the studies using the PRISMA flow diagram. LC: learning curve.

A total of 195 records were identified with our search strategy (Appendix A). After the removal of 87 duplicates, 108 articles were screened for eligibility (Figure 1). Of these, 92 abstracts were excluded, and sixteen full texts were assessed for inclusion. One of them had not performed a formal assessment of LC and was further excluded [15]. Finally, fifteen studies were included in the final meta-analysis [5,16,17,18,19,20,21,22,23,24,25,26,27,28,29].

### 3.1. Summary of Included Studies

Fifteen articles assessed the LC in pediatric RALP. Table 1 shows the summary of the included studies. The method of LC representation differed between the studies. The number of surgeons varied from single to multiple.

**Sorensen et al.** compared the LC and outcomes of 33 consecutive children undergoing RALP from 2006 to 2009 to open surgery [16]. Outcomes assessed were operative time, complications, postoperative pain, length of stay and surgical success for two surgeons who adopted the robotic approach. The authors found no significant differences in length of stay, pain score or surgical success at a median follow-up of 16 months. The number of complications was similar, and they tended to present early in the RALP group. After 15 to 20 robotic cases, the overall operative times for RALP cases were consistently within 1 SD of the average open pyeloplasty time. Of the decrease in overall operative time, 70% was due to decreased pyeloplasty time rather than peripheral time.

**Stern et al.** quantified the LC for a single surgeon based on a prospective cohort of 50 consecutive RALP patients using cumulative sum (CUSUM) analysis [17]. The outcomes assessed were total operative time (OT) and step-specific operative times for port placement, dissection and hitch stitch placement, pelvis dismemberment and spatulation, suturing, and port removal. There was a significant difference in the mean OT time between learning—the initial 13 cases (mean 203.9 min), proficiency—the middle 16 cases (mean 159.2 min), and competency—the last 21 cases (mean 126.6 min.) The complication rate stabilized around the acceptable level of 5%. The step-specific analysis suggested that suturing entered the competency phase at case 27, with a 50% decrease in suturing time from learning to proficiency and competency.

**Reinhardt et al.** compared the clinical outcome of the first 25 RALP performed by a single surgeon to the data on open and laparoscopic procedures from the previous 5-year period [18]. The median operating time in robotic surgery was 182 min and was significantly shorter than in laparoscopic surgery (250 min). The postoperative inpatient length of stay was significantly shorter after robotic surgery (median 1 day) than after both laparoscopic (median 2 days) and open surgery (median 3.5 days).

**Cundy et al.** assessed the LC using the CUSUM method in a total of 90 RALP cases [19]. The LC transitioned beyond the learning phase at cases 10, 15, 42, 57, and 58 for set-up time, docking time, console time, operating time, and total operating room time, respectively. All the comparisons of mean operating times between the learning phase and subsequent phases were statistically significant. However, no significant association was observed between case experience and frequency of post-operative complications.

**O’Brien et al.** reviewed 20 cases of RALP and compared them to children who had undergone laparoscopic and open pyeloplasties [20]. A gradual decrease in operative time in RALP cases was described. The length of hospitalization and postoperative analgesia requirements were greater in the age-similar open pyeloplasty group compared to the other two groups. Intraoperative times were greater in the laparoscopic and RALP groups compared to the open pyeloplasty group.

**Kassite et al.** analyzed the learning curve using cumulative sum (CUSUM) methodology for OT and a composite parameter (combination of three parameters: OT adjusted for patient complexity factors (AOT), complications, and surgical success) [5]. Two surgeons without any experience in robotic surgery performed 42 consecutive RALP in 41 patients. Based on the CUSUM analysis for composite outcome, the authors concluded that the learning curve for RALP could be divided into three different phases: phase 1, the learning period (1–12 cases); phase 2, the consolidation period (13–22 cases); and phase 3, representing the period of increased competence (23–39th case). A multi-outcome approach was adopted to provide a comprehensive view of the learning process for RALP. The authors recommend that more than 41 cases are needed to achieve mastery.

**Esposito et al.** analyzed the learning curve of RALP for the outcomes time for docking and anastomotic time in 28 consecutive procedures performed by the same robotic team (console surgeon, bedside surgeon, scrub nurses) and found that the docking time fell from 48 min to 15.5 min whereas the anastomotic time significantly decreased from 98 min to 49 min following 23 consecutive RALP procedures [21].

**Radford et al.** compared the LC for operative time with two suture subtypes and found more rapid reduction in the unidirectional barbed suture (V-Loc^®^) group as compared to the classic 5–0 Vicryl^®^ suture group [22]. The operative time reduced with the number of cases. A standard LC existed over 18 months. However, when comparing V-Loc and Vicryl cases performed around the same point of the curve in this study, the V-Loc cases were faster.

**Tasian et al.** evaluated the operative time of 4 pediatric urology fellows, finding an average decrease of 3.7 min per case [23]. Fellows were projected to achieve the median attending operative time after 37 cases. No operative complications or failed pyeloplasties occurred. They underlined that ascertaining LC would allow optimally structured fellowships and would enabling trainees to acquire the requisite skills before training is completed.

**Mason et al.** evaluated the effectiveness of the proctor environment on the LC of faculty pediatric urologists training to perform RALP, and compared procedures performed by an expert surgeon and two training surgeons with extensive prior laparoscopic experience [24]. The training surgeons were proctored for three cases before becoming independent operators.

**Murthy et al.** retrospectively reviewed a series of open pyeloplasties and RALP performed by a single surgeon [25]. Operative times were significantly longer for RALP (203.3 vs. 135.0 min) but a significant downward trend for operative time was demonstrated with increasing operative experience, reaching the mean operative time of the open procedure in the last ten RALP cases. Complications tended to occur early.

**Andolfi et al.** retrospectively reviewed pyeloplasty cases for the treatment of ureteropelvic junction obstruction (UPJO) in infants at three academic institutions [26]. LC of RALP were studied by r-to-z transformation and CUSUM, and were compared to open and laparoscopy groups. LC showed a plateau in OT after 18 cases for RALP and showed a second phase of further improvements after 37 cases. At 16 months follow-up, there were similar rates of success and complications between the three groups.

**Bowen et al.** reviewed pediatric RALP cases by three surgeons [27]. The authors analyzed the impact of proctoring and the impact of a dedicated robotics staff and program. The first aspect was defined by comparing the first eight cases of two surgeons transitioning from open to robotics, one without proctoring. The second aspect was analyzed by comparing the first ten cases of the fellowship-trained surgeon and the first and last eight cases of the robotic surgeon. Operative times were longer with an inexperienced robotics team. The proctored surgeon achieved shorter operative times more quickly. It was thus concluded that surgeons transitioning from open to robotic surgery can reach expertise levels in an established robotic surgical program of proctoring.

**Dothan et al.** retrospectively evaluated the data of all children who underwent pyeloplasty since 2003 in a single center [28]. The children were divided into three groups: open, laparoscopic and RALP. Each group was divided into two different phases: early and late. The median duration of surgery in the RALP group was significantly shorter than the open group (65 min vs. 72.5 min *p* < 0.01), while the first RALP case was already shorter than the median duration of surgery in OP group. There was no significant decrease in the duration of surgery in the RALP group over the study period. There was no difference in the length of stay in the early vs. late phases in the RALP group. There was no difference in the complications and success rate between the early and late phases of the RALP group. The authors concluded that previous experience in open and laparoscopic surgery may contribute to a shorter learning curve in robotic surgery.

**Junejo et al.** retrospectively reviewed fifteen patients with ureteropelvic junction obstruction (UPJO) who underwent RALP [29]. Of fifteen cases, nine were primary and six cases secondary UPJO. Total operative time was prolonged in secondary group as compared to the primary pyeloplasty group. The evaluation of the learning curve of RALP for this group of patients concluded that total operative time for RALP, performed by the pediatric urology team, steadily decreased with collective surgical experience.

### 3.2. Outcomes

Most studies had operative duration as the variable dependent on surgical volume and thus the measure of proficiency (Table 2). CUSUM was used for LC analysis in four studies [5,17,19,26], all others graphically depicted time vs. operative accumulation. The studies using CUSUM methodology identified changes in operative durations with more precision.

#### 3.2.1. Primary Outcomes

In all included studies, learning was assessed at least through operative times. In four studies [5,17,19,26], the operative times were further segmented into different phases of LC. Sorensen et al. [16] analyzed the surgical outcomes within different stages of the learning curve. No significant differences in surgical success, length of stay or pain scores were detected at a median follow-up of 16 months. All studies presented data on complications in relation to LC. None of the studies presented data on maintenance or decline of proficiency and competence stages of the LC. However, the study by Radford et al. [22] concluded that a standard learning curve existed over 18 months.

While all studies acknowledged complication rates, neither robotic, nor surgical competence were specifically defined in any of the studies except for those by Sorensen et al. [16] and Kassite et al. [5]. Sorensen et al. [16] estimated that 15 to 20 cases are needed for rudimentary proficiency when evaluating safety, efficacy, and operative time. Kassite et al. [5] defined competence through a composite score. With regards to complications and surgical success, RALP was safe and effective already during initiation, but cumulative case volumes improve efficiency.

In all studies, RALP was assessed to be safe and feasible with acceptable complication rates and expected outcomes of significant decreases in the degree of hydronephrosis were observed. Complications tended to occur early during LC, but no significant differences in complications or outcomes could be detected in the control groups.

#### 3.2.2. Secondary Outcomes

Confounding factors in the analysis of LC were seldom addressed in the included studies. Kassite et al. [5] utilized the CUSUM methodology for OT and a composite parameter was constructed for more precise proficiency assessment. There was not a clear unanimity in how to address LC despite all studies focusing on the operative durations. The definition of operative duration varied between studies.

### 3.3. Methodological Quality Assessment

Upon utilizing the Newcastle–Ottawa scale, only two studies [16,18] were rated as good quality. The rest all had a poor quality (Table 3). Except for those two studies, all studies were weaker in the comparability domain.

## 4. Discussion

Pediatric robot-assisted pyeloplasty is safe during the learning process with good outcomes, and ‘learning by doing’ improves efficiency. We summarized the current published data on learning curves in pediatric RALP. The analyzed dataset consisted of 723 operations and twenty-five robotic surgeons from eight different countries with articles published during 2011–2022 and their respective clinical material consisting of pediatric RALP from 2003 onwards. All RALPs were performed on the DaVinci platform (Intuitive Surgical, Sunnyvale, CA, USA). Most of the analyzed articles had the operative time or a derivative as the primary outcome measure for assessing learning.

Reaching surgical competency demands mastering many a skill, not limited to technical proficiency. Competency is composed of a triad of knowledge, skills, and attitudes [30]. The robotic surgeon must not only learn new skills in mastering the robot but must learn new components of attitudes as well. In robotic surgery, where the robotic surgeon is further away from the operating team, communicational skills must be honed. The current robots do not provide tactile feedback, which forces the robotic surgeon to learn a new manner of tactile vision where visual cues replace haptics. Thus, robotic surgeons and the whole team are faced with new settings in the operating room.

Adopted new technologies in surgery must be equal or superior to previous standards if practices are to be changed. Before a new technology or surgical method is validated, the surgeons must be competent in applying it to clinical practice. This has been measured by LC, especially within minimally invasive surgery. LC describe how new surgical skills are integrated and are commonly used to assess efficient surgical performance [31]. Experience curves as an organizing framework for deliberate practice in emergency medical learning. The use of LC started in the 1980s and has since been applied within most fields of medicine [32].

The most frequent variable for evaluating learning is operative time. However, the measured time interval is often poorly defined [33,34]. Although operative time segments can be clearly defined and thus analyzed, competence is much broader. It is of little value to record operative times if they are assessed without reference to surgical outcomes [23]. In surgery, precision outweighs speed for successful surgical outcome. As previously shown by Kassite et al. [35], the outcomes in studies assessing LC are heterogeneous and mostly focus on technical performance, neglecting broader competence and clinical outcomes.

Learning outcomes must be clearly defined and may vary between operations. LC also consist of different stages. Most studies focus on the initial curve, often omitting the expert plateau and the decline of competency [34,36,37,38]. Specific LC used to measure competence entail limitations if competence is not clearly defined. In our cohort, only four studies [5,17,19,26] clearly defined steps measured in robot-assisted pyeloplasty. The duration of surgery depends on many variables, not just on the difficulty of the surgery. Robotic pyeloplasty varies somewhat depending on previous medical history [23,39]. One may encounter adhesions after infections, percutaneous pyelostomies can lead to adhesions, and the rotation of the kidney varies between patients; for some, the kidney has to be reached transmesenterially, while in others, the colon needs to be released. Further, performing RALP highly depends on the team’s functioning, and the total operating time depends on the team’s effort [40]. Changes in local routines for double-J stent placement seem to vary, as some centers perform a cystoscopy before the robotic procedure, while others place the double-J stent intraoperatively, and some perform stentless RALP, further confounding the analysis of operative times. Suturing of the anastomosis can be regarded as solely console-surgeon dependent, and this was not analyzed in any of the included studies except for the study by Stern et al. [17]. The study by Kassite et al. [5] was the only one which controlled for patient complexity factors. They defined competency by a composite score which combined patient complexity factors, complications, and surgical success (patient adjusted operative time x complication factor x success factor).

In only two studies [17,26] was the proficiency addressed and estimated as occurring at 16 and 18 cases, respectively. The LC of competence with operative time as an outcome variable was assessed in seven studies estimated to occur at a mean of 31 cases [5,16,17,19,21,23,26]. Radford et al. [22] concluded that a standard LC existed for over 18 months. The inter-operation interval for maintaining proficiency or competency was not distinctly addressed in any of the included studies. Neither was the point of decreasing competency concerning diminishing numbers of operations discussed in any of the studies. CUSUM is a statistical process control tool for assessing consecutive performances, which may aid in detecting trends that may otherwise be undetected due to natural data variance [19]. CUSUM was used for LC analysis in four studies and considered to help identify breakpoints in LC [5,17,19,26]. While CUSUM enables a more accurate analysis of LC and is still under-utilized, defining competency remains the critical issue regarding learning robotic surgery.

Currently, the two available clinical robotic systems for pediatric use are the DaVinci^®^ (Intuitive Surgical) and Senhance^®^ (Transenterix) systems. All studies included analyzed surgeries performed on the DaVinci platform. Future studies will determine whether different platforms will influence outcome measures, LC, and learning robot-assisted surgery in general.

Pediatric urology entails many diagnoses, but the number of distinct operations with a given diagnosis per surgeon is usually limited. A pediatric urologist is rarely exposed to high volumes of a given operation but, on the other hand, may have experience from synchronous operations. Measuring LC for pediatric urology therefore faces challenges and should not be limited to only the initial phase. Understanding the variables of the LC is necessary when comparing operative times between surgeons, surgical teams, and surgical centers.

When initiating a robot curriculum for surgeons-in-training, it is imperative to define competencies and variables to be measured within them. The DaVinci^®^ platform mandates a robotic curriculum before initiating clinical activities. This consists of knowledge quizzes, assessing technical proficiency in robot handling, and liaising with the robotic team. Thus, a robotic fellowship must include structured training with all components of competency addressed. In the studies analyzed here, the respective training programs were not touched upon, but due to requirements from the robot providers, it is assumed that at least the minimal requirements were fulfilled. Only two studies addressed the role of proctoring and concluded that it expedites learning [24,27]. Learning by proctor instruction enhanced and shortened the LC; however, the outcome analyzed was only operative duration. In the study of Tasian et al. [23], structural fellowships were suggested, enabling trainees to acquire the requisite proficiency prior to the completion of training.

There are a few limitations for the present study. First, the definitions and cut-offs for attaining proficiency and competency in LC are not standardized, and are therefore highly subjective. Second, the published literature is of poor methodological quality and mostly single surgeon, single center observations, with relatively small sample size. Third, the prior experience of the surgeons differed amongst the included studies. Fourth, only a few studies have compared the data of LC of pRALP with open or laparoscopic surgery. Fifth, the assessment of the LC was widely variable. CUSUM approach, which is considered as the superior method of assessing LC, was used in only four of the included studies. Sixth, there was incomplete reporting of patient characteristics in the individual studies, leading to variability in surgical settings and confounding LC. Seventh, the operative approach was incompletely reported in the studies; in ten, the kidney was approached transperitoneally; however, the approach was not clearly presented in five studies. While this research is limited by the aspects mentioned above, including incomplete data reporting in individual studies, future research will embrace these shortcomings.

## 5. Conclusions

Competencies within robot-assisted surgery must be defined and related to surgical outcomes. The current mode of assessing LC by operative duration or simple technical tasks must be replaced by competencies in future studies. As pediatric RALP is safe already during initial phases, robotic fellowships may be introduced during residency. The training program should include different instructional designs adapted to the individual surgeon in line with modern surgical learning.

## Figures and Tables

**Table 1:** Baseline characteristics of the included studies.

Year of Publication	Author	Journal	Country	Sample Size	No. of Surgeons	Study Design
2011	Sorensen et al. [16]	J Urol	USA	33	2	Retro
2012	O’Brien et al. [20]	J Pediatr Urol	USA	20	1	Retro
2013	Tasian at al. [23]	J Urol	USA	100	5	Pro
2014	Mason et al. [24]	J Robotic Surg	USA	134	3	Retro
2015	Cundy et al. [19]	J Pediatr Surg	UK	90	1	Pro
2015	Murthy et al. [25]	Ann R Coll Surg Engl	USA	52	1	Retro
2016	Bowen et al. [27]	J Robot Surg	USA	28	3	Retro
2017	Reinhardt et al. [18]	Scandinavian J Urol	Denmark	25	1	Pro
2017	Radford et al. [22]	J Laparoendosc Adv Surg Tech A	UK	25	NA	Retro
2018	Kassite et al. [5]	J Pediatr Urol	France	42	2	Pro
2019	Esposito et al. [21]	J Pediatr Urol	Italy	37	3	Retro
2019	Junejo et al. [29]	Urol Ann	Saudi Arabia	15	NA	Retro
2020	Dothan et al. [28]	J Robot Surg	Israel	33	1	Retro
2022	Stern et al. [17]	J Pediatr Urol	Canada	50	1	Pro
2022	Andolfi et al. [26]	World J Urol	USA	39	1	Retro

Abbreviations: Retro, retrospective. Pro, prospective. USA, United States of America. UK, United Kingdom. NA, not applicable.

**Table 2:** Details of learning curves in the included studies.

Year of Publication	Author	LC Presentation	LC Outcomes	LC Comparison with Open/Laparoscopy	LC Case Number
2011	Sorensen et al. [16]	Narrative, line graph	Total operative time; postoperative complications	Open	15 to 20
2012	O’Brien et al. [20]	Narrative, line graph	Total operative time	Laparoscopy, Open	NA
2013	Tasian at al. [23]	Narrative, plot graph	Console time, intraoperative complications, resolution	No	37
2014	Mason et al. [24]	Narrative, line graph	Total operative time, intraoperative complications, postoperative complications, length of hospital stay	No	3
2015	Cundy et al. [19]	CUSUM chart, narrative, line graph, plot graph	Set up time, docking time, console time, operating time, total operating room time, postoperative complications	No	LC transitioned beyond the learning phase at cases 10, 15, 42, 57, and 58 for set-up time, docking time, console time, operating time, and total operating room time, respectively
2015	Murthy et al. [25]	Narrative, plot graph	Total operative time, intraoperative complications	Open	42
2017	Bowen et al. [27]	Narrative, line graph	Total operative time, intraoperative complications, postoperative complications, length of hospital stay, resolution	No	
2017	Reinhardt et al. [18]	Narrative, line graph	Total operative time, length of hospital stay, complications	Laparoscopy, Open	NA
2017	Radford et al. [22]	Narrative, plot graph	Operative time	No	NA
2018	Kassite et al. [5]	CUSUM chart	Operative time, adjusted operative time, composite parameter (operative time adjusted for patient complexity factors, complications factor and success factor)	No	41
2019	Esposito et al. [21]	Narrative, line graph	Time for docking and anastomosis duration	Laparoscopy	23
2019	Junejo et al. [29]	Narrative, line graph, table	Total operation duration, length of stay, complications, resolution	No	15
2021	Dothan et al. [28]	Narrative	Total operation duration, length of stay, complications, resolution	Laparoscopy, Open	NA
2022	Stern et al. [17]	Narrative, CUSUM chart	Total operative time, step-specific operative times for port placement, dissection, and hitch stitch placement, pelvis dismemberment, and spatulation, suturing and port removal	No	Learning—initial 13 cases, proficiency—middle 16 cases, competency—last 21 cases
2022	Andolfi et al. [26]	r-to-z transformation, CUSUM	Total operation duration, complications, resolution	Laparoscopy, Open	LC showed plateau in OT after 13 cases and a second phase of further improvements after 37 cases

Abbreviations: LC, learning curve. CUSUM, cumulative sum analysis. NA, not applicable. OT, operation theatre.

**Table 3:** Methodological quality assessment utilizing the Newcastle–Ottawa scale.

Author, Year	Selection				Comparability	Outcome			Total Score	Quality
	Item 1	Item 2	Item 3	Item 4	Item 5	Item 6	Item 7	Item 8		
Sorensen et al., 2011 [16]	*	*	*	*	*	*	*	*	8	Good
O’Brien et al., 2012 [20]	*	*	*	*	-	*	*	*	7	Poor
Tasian at al., 2013 [23]	*	-	*	*	-	*	*	*	6	Poor
Mason et al., 2014 [24]	*	-	*	*	-	*	-	*	5	Poor
Cundy et al., 2015 [19]	*	-	*	*	-	*	*	*	6	Poor
Murthy et al., 2015 [25]	*	*	*	*	-	*	*	*	7	Poor
Bowen et al., 2017 [27]	*	-	*	*	-	*	*	*	6	Poor
Reinhardt et al., 2017 [18]	*	*	*	*	*	*	*	*	8	Good
Radford et al., 2017 [22]	*	-	*	*	-	*	*	*	6	Poor
Kassite et al., 2018 [5]	*	-	*	*	-	*	*	*	6	Poor
Esposito et al., 2019 [21]	*	*	*	*	-	*	*	*	7	Poor
Junejo et al., 2019 [29]	*	-	*	*	-	*	*	*	6	Poor
Dothan et al., 2021 [28]	*	*	*	*	-	*	-	*	6	Poor
Stern et al., 2022 [17]	*	-	*	*	-	*	*	*	6	Poor
Andolfi et al., 2022 [26]	*	*	*	*	-	*	*	*	7	Poor

Good quality: 3 or 4 stars in selection domain AND 1 or 2 stars in the comparability domain AND 2 or 3 stars in the outcome domain. Poor quality: 0 or 1 star(s) in selection domain OR 0 stars in the comparability domain OR 0 or 1 star(s) in the outcome domain.

## Data Availability

Study data are available from authors on reasonable request.

## Appendix A

**PubMed**—(learning curve) AND (pediatric pyeloplasty).

**Embase**—‘learning curve’:ti,ab,kw AND ‘pediatric pyeloplasty’:ti,ab,kw.

**Scopus**—TITLE-ABS-KEY (learning curve) AND TITLE-ABS-KEY (pediatric pyeloplasty).

**Web of Science**—Query 1: ALL = (learning curve) Query 2: ALL = (pediatric pyeloplasty).

**Database**	**Studies**
PubMed	54
Embase	6
Scopus	68
Web of Science	67
Total	195
Duplications	87
After duplications removal	108
